# Immunohistochemical localization of progesterone receptors alpha (PRA) in ovary of the pseudopregnant rabbit

**DOI:** 10.21451/1984-3143-AR2018-0128

**Published:** 2019-10-24

**Authors:** Mahmoud Abd-Elkareem, Alaa Sayed Abou-Elhamd

**Affiliations:** 1 Department of Anatomy, Histology and Embryology, Faculty of Veterinary Medicine, Assiut University, Egypt.; 2 Department of Medical Laboratory Technology, Faculty of Medical applied sciences, Jazan University, KSA.

**Keywords:** Ovary, progesterone, pseudopregnancy, rabbit

## Abstract

Progesterone plays an important role in the reproductive function and follicular development in mammals. The aim of the present study was to examine the localization of progesterone receptor alpha (PRA) in ovary of pseudopregnant rabbit by immunohistochemical methods. Samples were collected from 14 h. to 18 days of pseudopregnancy. At the first stage of pseudopregnancy (14 h.), the rabbit ovary showed moderate immunostaining of PRA in the granulosa cells and theca interna cells of preovulatory follicle and in the stroma cells. At the middle stage of pseudopregnancy (3-7 days), the rabbit ovary showed strong immunostaining of PRA in ovarian surface epithelial cells, follicular cells of the primary follicle, granulosa cells and theca interna cells of the growing and antral follicles. Moderate immunoexpression of PRA were observed in the large lutein cells and endothelial cells of the corpus haemorrhagicum and corpus luteum and in the stroma cells. At the end of pseudopregnancy (18 days) strong PRA reactions were detected in the small lutein cells of the regressed corpus luteum. Moderate to strong PRA immuno-expression were observed in the proliferated theca interna cells of the atretic antral follicles. The atretic large lutein cells of the regressed corpus luteum showed negative immunostaining for PRA. This study showed that the PRA positive small lutein cells of the regressed corpus luteum and the PRA positive proliferated theca interna cells of the atretic antral follicles were transformed into PRA positive interstitial gland cells. In conclusion, the present study had described the distribution of PRA in the ovary of pseudopregnant rabbit, which is not discussed before in the available literature. It also gives more information about follicular dynamic, formation and origin of interstitial glands, mechanism of ovulation, formation and regression of the corpus luteum.

## Introduction

Progesterone (P_4_) is a steroid hormone that plays an important role in female reproductive activity in vertebrates such as regulation of follicular development, growth and differentiation of ovarian structures, ovulation and luteinization. It also participates in maintenance of pregnancy and breast development (Spencer and Bazer, 2002; Conneely *et al*., 2003; Anzaldua Arce *et al*., 2010). It is well known that the effects of P4 are mediated by its interaction with specific hormone-binding proteins called progesterone receptor (PR). Progesterone receptor is belonging to the nuclear receptor superfamily that encoded by single gene which consists mainly of two different isoforms; progesterone receptors alpha (PRA) and progesterone receptors beta (PRB) (Conneely *et al*., 2002, Kubota *et al*., 2016).The amount of these receptors is under hormonal control (Blauer *et al*., 2005; Anzaldua Arce *et al*., 2010). They up-regulated by estradiol and down-regulated by progesterone (Kraus and Katzenellenbogen, 1993; Anzaldua Arce *et al*., 2010; Camacho-Arroyo *et al*., 2003). Immunohistochemical localization of PR has been described in reproductive organs of mouse and human (Teilmann *et al*., 2006), ovaries of monkey, human, bovine and rabbit (Hild-Petito *et al*., 1988; Revelli *et al*., 1996; D'Haeseleer *et al*., 2007; Abd-Elkareem, 2017a) respectively and uterus of rat and pseudopregnant rabbit (Hegele-Hartung *et al*., 1992; Kraus and Katzenellenbogen, 1993; Abd-Elkareem, 2017a).

In our previous study (Abd-Elkareem, 2017a) we demonstrated the localization of PRA in rabbit ovary during pregnancy up to 10 days after pregnancy. The distribution of PRA in the ovary of pseudopregnant rabbit has not been discussed before in the literatures. Therefore, we carried out this study to demonstrate the immunolocalization of PRA in the ovary of pseudopregnant rabbit at different periods starting from 14hr to 18 days of pseudopregnancy. In addition to characterize the different levels of PRA expression in rabbit ovaries from 14 h to 18 days.

## Materials and Methods

### Animals and tissue collection

Thirteen mature female New Zealand white rabbits (2.4 ± 0.06 Kg body weight and 4- 5 months old) were housed in separate cages under 22-25 ºC room temperature and controlled light (12 h light/ dark cycle) conditions.

Rabbits were induced to ovulate by intramuscular injection of HCG (50- 70 IU Choriomon, IBSA Institute Biochimique S.A., Lugano, Switzerland). The day of induction was considered as 0 days of pseudopregnancy.

The protocol used in this experiment was approved by the Committees of use and care of experimental animals of Faculty of Veterinary Medicine, Assiut University, Egypt.

### Sampling

Right and left ovaries were collected at 14 h, 3, 7 and 18-days post-induction of ovulation. For each experimental period, ovaries of two to three animals were dissected immediately after slaughtering, and then they fixed with Bouin’s fluid.

### Immunohistochemistry

The fixed samples were processed for paraffin embedding. 3-5µm sections were prepared from paraffin embedded samples, then they dewaxed in xylene and rehydrated in descending grades of alcohol. Sections were rinsed in PBS (pH 7.4) three times 5 min each. According to Abd-Elkareem (2017a), the slides were incubated successively in 3% hydrogen peroxide for 10 min at room temperature, PBS (pH 7.4four times 5 min each). For antigen retrieval, the slides were boiled in 10 mM sodium citrate buffer (pH6.0) 20 min followed by cooling for 20 min at room temperature then rinsed in PBS (pH 7.4 three times 1 min each time). Immunohistochemical detection of PRA was performed using PR (Clone SP2) and an Ultravision Detection System (Anti-Polyvalent, HRP/DAB; Thermo Fisher Scientific, USA). To inhibit the nonspecific background, sections were covered with Ultra V block for 5 min at room temperature. Sections were incubated with rabbit monoclonal antibody (1: 300) (Cat.#RM-9102S0, Thermo Fisher Scientific, USA) for 30 min at room temperature, then were washed with PBS at pH 7.4 (four times 5 min each). For detection of the primary antibody, sections were then incubated with a biotinylated anti-Rabbit antibody (1:300), Thermo Fisher Scientific, USA) for10 min at room temperature. Incubation was followed by three 5 min washes in PBS (pH 7.4) then the sections were incubated streptavidin- peroxidase complex (Thermo Fisher Scientific, USA) for 10 min at room temperature. Four times washes 5 min in PBS. Peroxidase activity was visualized by 5–15 min incubation at room temperature in a solution consisting of one drop of DAB (diaminobenzidine) Plus chromogen to 2 ml of DAB Plus substrate. Sections were counterstained in Harris hematoxylin. After washing in distilled water, the sections were dehydrated and cover slipped with DPX. Negative controls were performed as the previous steps without adding the primary antibody. Sections were examined using an OLYMPUS BX51microscope and photographed with an OLYMPUSDP72 camera adapted to the microscope. The assessment of intensity of the immunostaining in the nucleus and cytoplasm was dependent on its color: dark brown to black (strong or intense), brown (moderate), light brown (weak) and no immunoreactivity (negative immunostaining).

## Results

**Figure 1:**
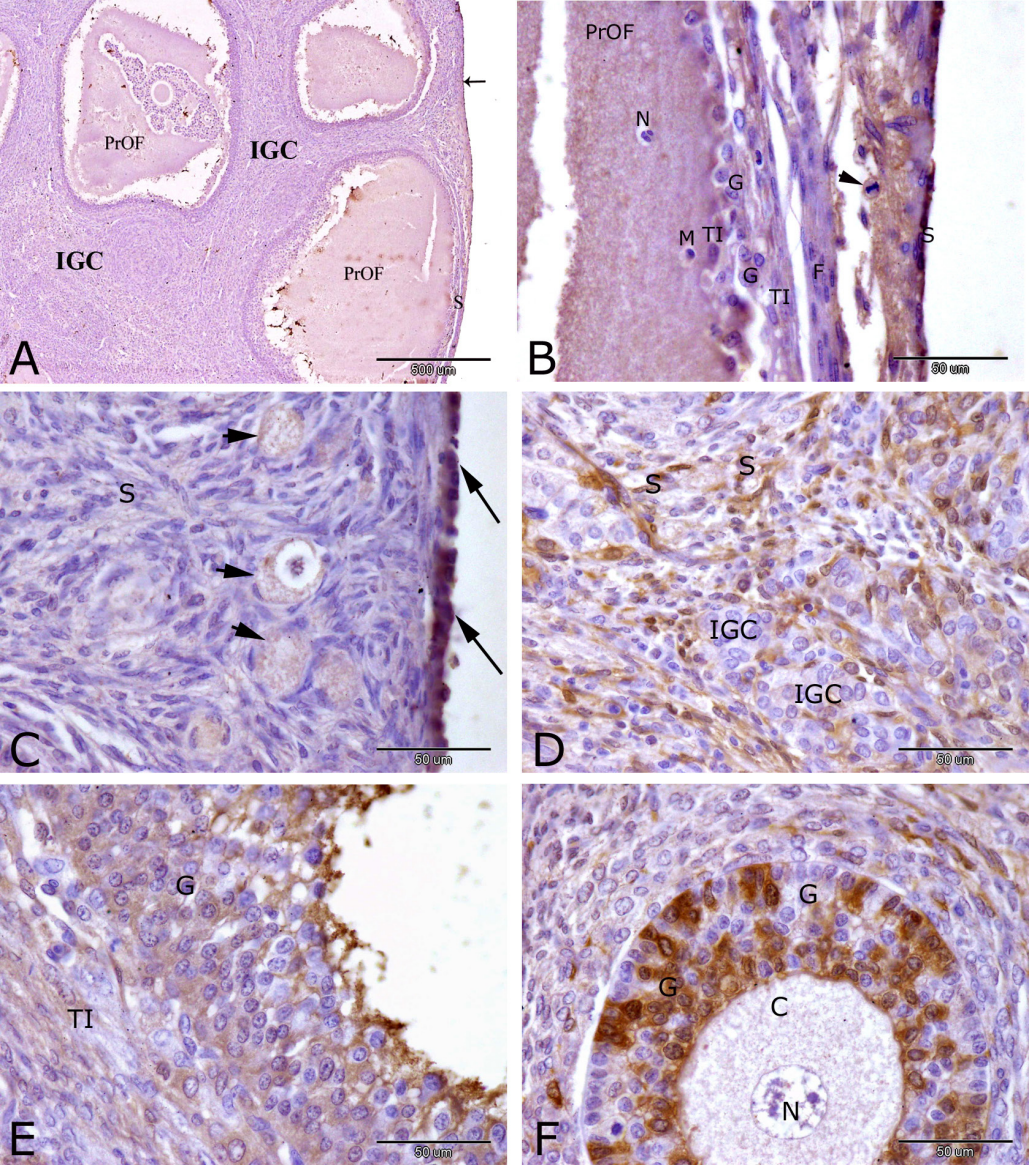
Immunostaining of progesterone receptor alpha (PRA) in the rabbit ovary at 14 hrs of pseudopregnancy. Moderate immunostaining of PRA was observed in ovarian surface epithelial cells (arrows, A and C) and granulosa cells of secondary (growing) follicles (G, F). Weak to moderate PRA reactivity were observed in preovulatory follicle (PrOF, A), interstitial gland cells (IGC, A), cytoplasm of oocytes (arrowheads, A) of primordial follicles, granulosa cells (G) and theca interna cells (TI) of preovulatory follicle (E) and in stromal cells (S, C and D). Negative immunostaining of PRA was observed in some interstitial gland cells (IGC, D), in the nucleus (N) and cytoplasm (C) of oocyte of growing follicles (F). Note stigma formation (S, A) the area of the ovarian surface where the preovulatory follicle (PrOF) will burst through during ovulation. Stigma in rabbit (B) contained weak to moderate PRA immunostaining in ovarian surface epithelial cells (S), macrophage (M), neutrophils (N), fibroblasts (F), mitotic figure of endothelial cells (arrowhead), intermingled granulosa cells (G) and theca interna cells (TI). Original magnification; A: X 40, scale bar = 500 µm and B, C, D, E and F: X 400, scale bar = 50 µm.

At 14 h of pseudopregnancy, immunohistochemical localization of receptors alpha (PRA) in the rabbit ovary showed moderate nuclear and cytoplasmic immunostaining of PRA in ovarian surface epithelial cells (Fig. 1A and C) and granulosa cells of secondary follicles (Fig. 1F). Weak to moderate reaction of PRA were observed in cytoplasm of oocytes of the primordial follicles (Fig. 1 C), in the interstitial gland cells (Fig. 1A), in the stromal cells (Figs. 1C and D), granulosa cells and theca interna cells of preovulatory follicle (Fig. 1E). Negative immunostaining of PRA was observed in some interstitial gland cells (1D), in the nucleus and cytoplasm of oocyte of secondary follicles (1F). Ovulation stigma could be demonstrated at this stage of pseudopregnancy in rabbits. It was the thinnest area of the ovarian surface where the preovulatory follicle will burst through during ovulation. Stigma in rabbit contained weak to moderate PRA immunostaining in the ovarian surface epithelial cells, macrophage, neutrophils, fibroblasts, intermingled granulosa cells and theca interna cells and in the mitotic figure of endothelial cells.

**Figure 2:**
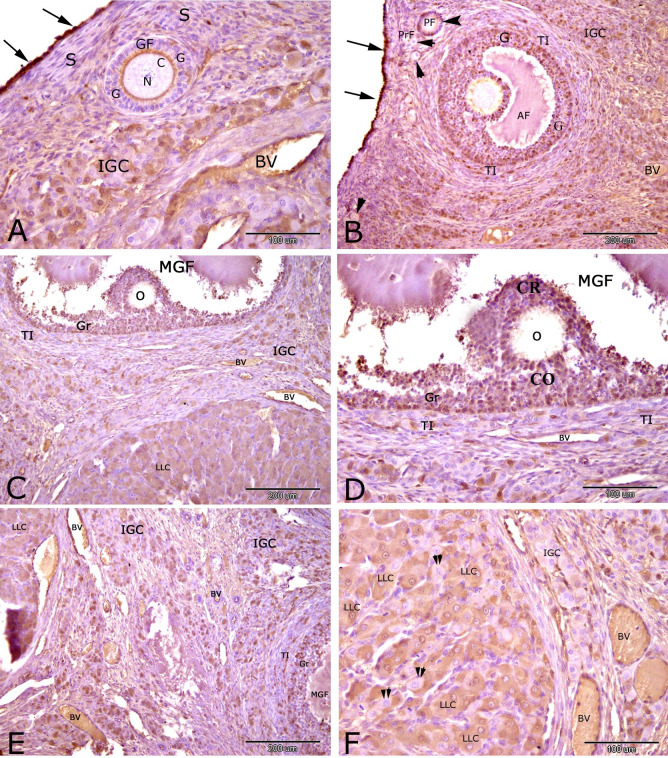
Immunostaining of progesterone receptor alpha (PRA) in the rabbit ovary at 3 days of pseudopregnancy. Strong immunostaining of PRA was observed in ovarian surface epithelial cells (arrows, A and B). Moderate immunostaining of PRA was observed in follicular cells (arrowhead) of the primordial (PrF, B) and primary (PF, B) follicles, in zona pellucida (arrow head) of the growing follicle (GF, A), in granulosa cells (G) and theca interna cells (TI) of the antral follicle (AF, B) andof the mature Graafian follicles (MGF, C, D and E), in corona radiate (CR) and cumulus oophorus (CO) of the mature Graafian follicles (MGF, D), inthe interstitial gland cells (IGC, A, C, E and F), in the endothelial cells (E) of the blood vessels (BV) and in large lutein cells of corpus luteum (LLC, C and F). Negative immunostaining of PRA was observed in some large lutein cells of corpus luteum (Two arrow heads, F), in the granulosa cells (G) of the growing follicle (GF, A), in the nucleus (N) and cytoplasm (C) of oocytes (O) of primordial,primary, growing and mature follicles (A, B, C and D) and in the stromal cells (S, A).Original magnification; A, D and F: X 200, scale bar = 100 µm and B, C and E: X 100, scale bar = 200 µm.

At 3 days of pseudopregnancy, the rabbit ovary showed strong nuclear and cytoplasmic immunostaining of PRA in ovarian surface epithelial cells (Figs. 2A and B). Moderate nuclear and cytoplasmic immunostaining of PRA were observed in follicular cells of the primordial and primary follicles (Fig. 2B), in zona pellucida of the growing follicle (Fig. 2A), in granulosa cells and theca interna cells of the antral follicle (Fig. 2B). Moderate reaction was also observed in granulosa cells, theca interna cells, corona radiata cells and cumulus oophorus cells of the mature Graafian follicles (Figs. 2C, D and E). Interstitial gland cells (Figs. 2A, C, E and F), endothelial cells of the blood vessels (Figs. 2A, C, D and E) and large lutein cells of corpus luteum (Figs. 2C and F) were expressed moderate PRA immunostaining. Negative immunostaining of PRA was observed in the granulosa cells (G) of the growing follicle (Fig. 2A), the nucleus and cytoplasm of oocytes of primordial, primary, growing and mature follicles (Figs. 2A, B, C and D) and in the stromal cells (2A).

**Figure 3:**
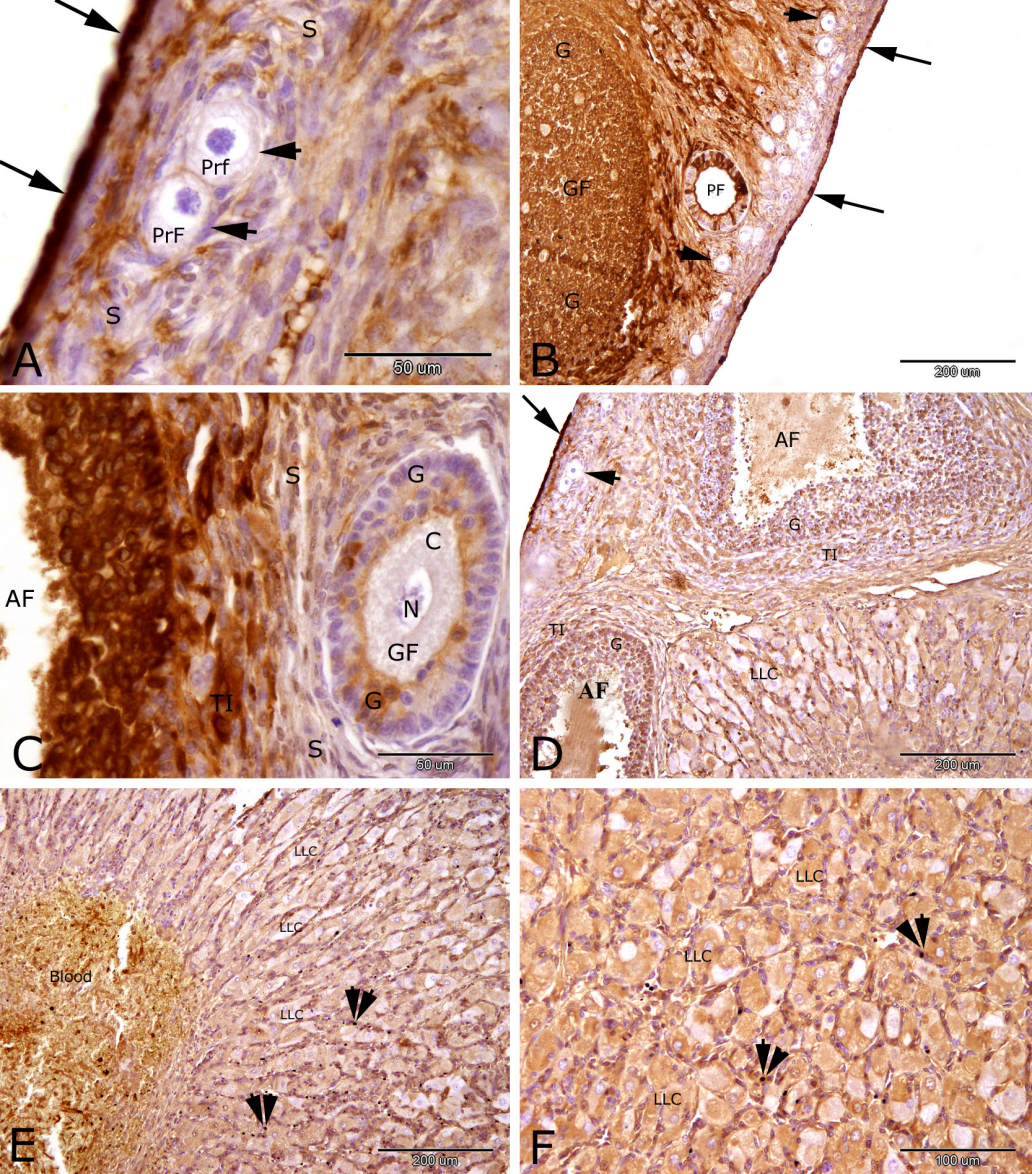
Immunostaining of progesterone receptor alpha (PRA) in the rabbit ovary at 7 days of pseudopregnancy. Strong immunostaining of PRA was observed in the ovarian surface epithelial cells (arrows, A, B and D), follicular cells (F) of the primary follicle (PF, B), granulosa cells (G) and theca interna cells (TI) of the growing follicles (GF, B) and antral follicles (AF, C). Moderate PRA immunostaining was observed in the granulosa cells (G) and theca interna cells (TI) of the growing (GF, C) and antral (AF, D) follicles, large lutein cells (LLC) and endothelial cells (double arrow arrowheads)of the corpus hemoragicum (E) and large lutein cells (LLC) and endothelial cells (double arrow arrowheads)of the corpus luteum (CL) (D and F), and in the stroma cells (S, A and C). Negative immunostaining of PRA was observed in the nucleus (N) and cytoplasm (C) of oocytes (O) of the primordial follicle (PrF, A), primary follicle (PF, B) and growing follicles (GF, C), follicular cells of the primordial follicle (arrowheads, A and B). Original magnification; A and C: X 400, scale bar = 50 µm, B, D and E: X 100, scale bar = 200 µm and F: X 200, scale bar = 100 µm.

By 7 days of pseudopregnancy, expression of PRA in rabbit ovary was strongly detected in the nucleus and cytoplasm of ovarian surface epithelial cells (Figs. 3A, B and D) follicular cells of the primary follicle (Fig. 3B), granulosa cells and theca interna cells of the growing follicles (Fig. 3D) and antral follicles (Fig. 3C). Moderate nuclear and cytoplasmic PRA reaction was observed in the granulosa cells and theca interna cells of the growing and antral follicles (Figs. 3C and D), large lutein cells and endothelial cells of the corpus haemorrhagicum (Fig. 3E) and large lutein cells and endothelial cells of the corpus luteum (Figs. 3D and F), and in the stroma cells (Figs. 3A and C). Negative immunostaining for PRA was observed in the nucleus and cytoplasm of oocytes of the primordial follicle (Fig. 3A), primary follicle (Fig. 3B) and growing follicles (Fig. 3C), follicular cells of the primordial follicle (Figs. 3A and B).

**Figure 4:**
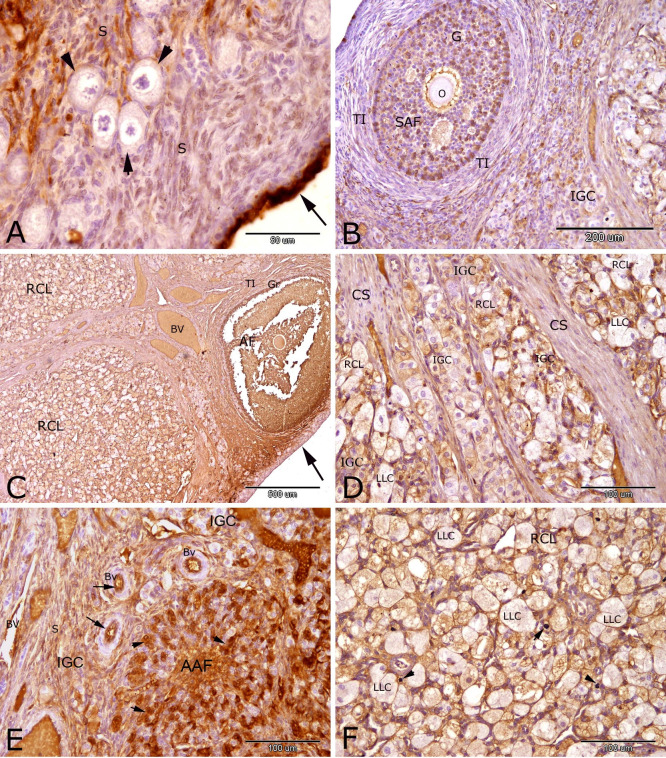
Immunostaining of progesterone receptor alpha (PRA) in the rabbit ovary at 18 days of pseudopregnancy. Strong reactions of PRA was observed in the ovarian surface epithelial cells (arrows, A) and in the small lutein cells (arrow head) of the regressed corpus luteum (F).Moderate to strong PRA immuno-expression were observed in the granulosa cells (G) of the small antral follicle (SAF, B), endothelial cells (arrow) of the blood vessels (Bv, E) and in the proliferated theca interna cells (arrow head) of the atretic antral follicles (AAF, E). Moderate reaction of PRA was observed in stromal cells (S, A and E) and in the granulosa cells (G) and theca interna cells (TI) of the large antral follicle (AF, C). Moderate PRA reaction was observed in the oocyte of the large antral follicle (O, C). Weak PRA reaction was observed in the oocytes of the primordial follicle (arrow heads, A), small antral follicle (O, B) and in the interstitial gland cells (IGC, B, D and E). Negative PRA immunostaining were observed in the large lutein cells (LLC) of the regressed corpus luteum (RCL, C, D and F). Note the proliferation of connective tissue to form numerous connective tissue septa (CS, D). Original magnification; A: X 400, scale bar = 50 µm, B: X 100, scale bar = 200 µm, C:X 40, scale bar = 500 µmand D, E and F: X 200, scale bar = 100 µm.

Immunostaining of PRA in the rabbit ovary at 18 days of pseudopregnancy showed strong nuclear and cytoplasmic reactions in the ovarian surface epithelial cells (Fig. 4A) and in the small lutein cells of the regressed corpus luteum (Fig. 4F). Moderate to strong PRA immuno-expression were observed in the granulosa cells of the small antral follicle (Fig. 4 B), endothelial cells of the blood vessels (Fig. 4E) and in the proliferated theca interna cells of the atretic antral follicles (Fig. 4E). Moderate reaction of PRA was observed in stromal cells (Fig. 4E) and in the granulosa cells and theca interna cells of the large antral follicle (Fig. 4C). Moderate PRA expression was observed in the oocyte of the large antral follicle (Fig. 4C). Weak PRA reaction was observed in the oocytes of the primordial follicle (Fig. 4A), small antral follicle (Fig. 4B). and in the interstitial gland cells (Figs. 4 B, D and E). Negative PRA immunostaining were observed in the large lutein cells of the regressed corpus luteum (Fig. 4 C, D and F). In this stage of pseudopregnancy the connective tissue of the regressed corpus luteum was proliferated to divide the regressed corpus luteum into complete separate lobules each of which contained regressed lutein tissue (Fig. 4D). The regressed lutein tissue was formed of atretic large lutein cells (PRA negative) and healthy small lutein cells (strong PRA immunostaining) which transformed into interstitial gland cells. This explained why the interstitial gland was formed of several lobules.

**Figure 5:**
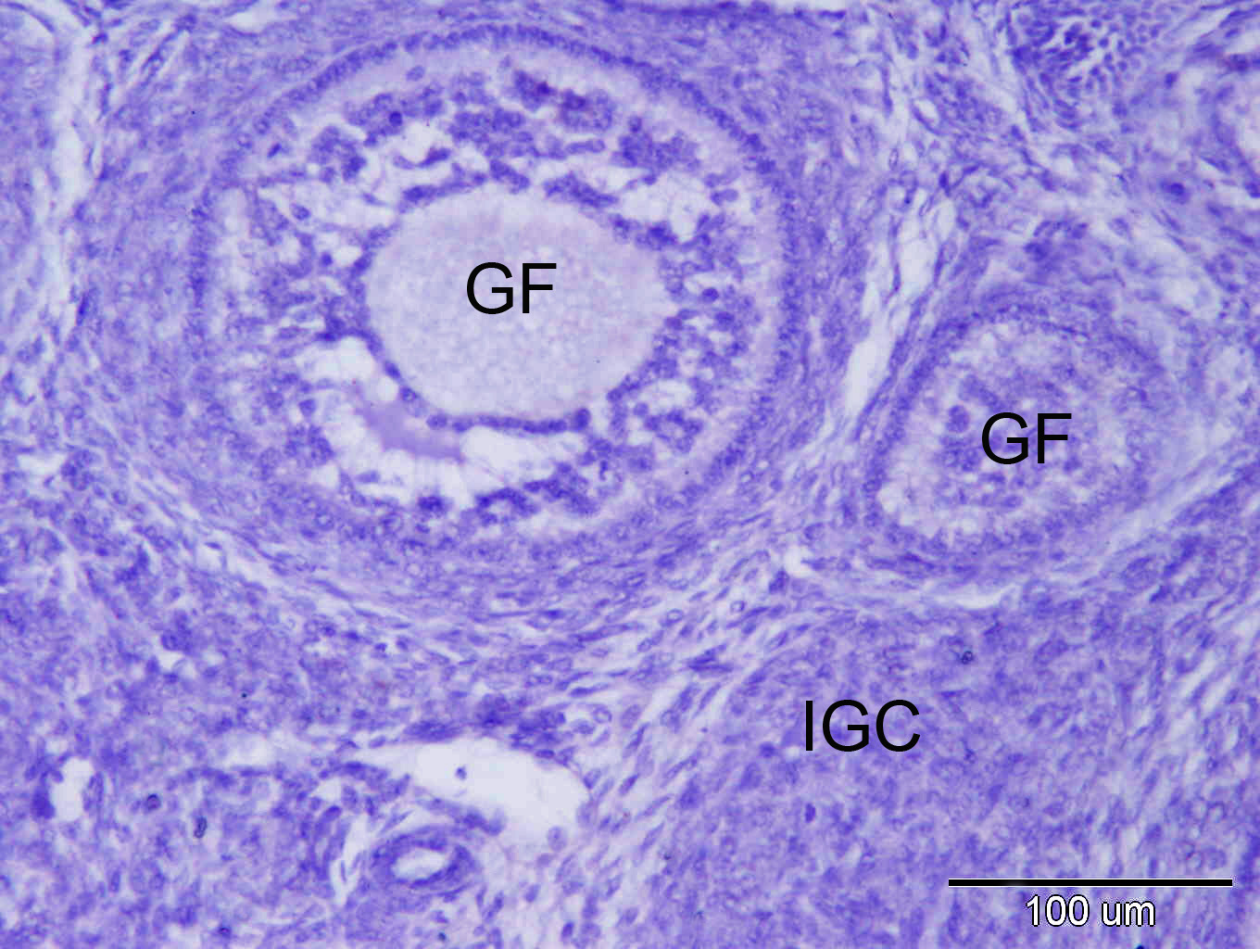
Control negative for PRA immunostaining in the rabbit ovary; note the growing follicles (GF) and the interstitial gland cells. (IGC). Original magnification; X 200, scale bar = 100 µm.

## Discussion

At the first stage of pseudopregnancy (14 h), immunohistochemical localization of progesterone receptors alpha (PRA) in the rabbit ovary showed moderate immunostaining of PRA in the granulosa cells, theca interna cells of preovulatory follicles, ovarian surface epithelial cells and in the stroma cells. These results are in accordance with findings in human (Jacobs *et al*., 1980; Iwai *et al*., 1990), primates (Hild-Petito *et al*., 1988), cattle (Van den Broeck *et al*., 2002; D'Haeseleer *et al*., 2007), pig (Slomczynska *et al*., 2000; Durlej *et al*., 2010), dog (Vermeirsch *et al*., 2001), rodent (Park-Sarge *et al*., 1995; Hulas-Stasiak and Gawron, 2010) and rabbit (Korte and Isola, 1988; Iwai *et al*., 1991; Parillo *et al*., 2013; Lan *et al*., 2014; Abd-Elkareem, 2017a).

Ovulation stigma could be demonstrated at 14 h of pseudopregnancy in rabbits. It was the thinnest areas of the ovarian surface where the preovulatory follicles will burst through during ovulation. It showed weak to moderate PRA immunostaining in the ovarian surface epithelial cells, macrophages, neutrophils, fibroblasts, granulosa cells and theca interna cells. PRA is essential for ovulation because mice lacking PRA don’t ovulate and are infertile (Gava *et al*., 2004; Mulac-Jericevic and Conneely, 2004). ADAMTS-1 (A disintegrin and metalloproteinase with thrombospondin-like motifs) and cathepsin L (a lysosomal cysteine protease) are two proteases induced in highest levels after LH stimulation in granulosa cells of preovulatory follicles in a PR-dependent manner (Robker *et al*., 2000), they are involved in degradation of the follicular wall. It was reported that the cells of the ovine ovarian surface epithelium are enzymatically involved in the ovulatory process by the influence of progesterone and its receptors (Murdoch, 1998). This indicated that the progesterone and its receptors (PRA) play a central role in the formation of ovarian stigma in pseudopregnant rabbit and this occurred by the action of immune cells (macrophages and neutrophils), connective tissue cells (fibroblasts) and ovarian cells (granulosa and theca interna cells). LH surge induces expression of PR by granulosa cells and withdrawal from the cell cycle. Progesterone by binding to progesterone receptor inhibits apoptosis. All these events promote resistance to apoptosis in the granulosa cells of bovine preovulatory follicles (Quirk *et al*., 2004).

At the middle stage of pseudopregnancy (3-7 days), the rabbit ovary showed strong immunostaining of PRA in ovarian surface epithelial cells, in follicular cells of the primary follicle and in the granulosa cells and theca interna cells of the growing and antral follicles. These data are in accordance with findings in pregnant and pseudopregnant rabbits (Korte and Isola, 1988; Iwai *et al*., 1991; Parillo *et al*., 2013; Lan *et al*., 2014; Abd-Elkareem, 2017a). Progesterone (P4) plays an important role in the intraovarian regulation of follicular growth and development (Revelli *et al*., 1996; D'Haeseleer *et al*., 2007). Progesterone promotes all stages of follicular development and directly suppresses the final large follicular stages only (Setty and Mills, 1987). The effects of P4 are mediated by its binding with specific progesterone receptor (PR). In bovine ovary, P4 regulate follicular development through the interaction of granulosa cells, theca cells, and stroma cells (D'Haeseleer *et al*., 2007). Granulosa cells, thecal/ stromal cells secrete P4 at different levels (Peluso, 2006) and they may have a different PRA immunoexpression pattern during pseudopregnancy. The expression of PR in granulosa cells was regulated by the activities of hCG (Iwai *et al*., 1991).

The expression of PRA in thecal cells indicated that thecal cells may mediate some actions of steroid hormones on the follicle cells in growing and antral follicles (Vermeirsch *et al*., 2001). As estradiol is the principle luteotropin in rabbits (Keyes and Armstrong, 1968) and only follicles make a significant amount of estrogen (Mills and Savard, 1973), follicular development and steroidogenic activity is essential for continuation of the corpus luteum in the pseudopregnant rabbit.

The present study showed moderate PRA immunostaining in the large lutein cells and endothelial cells of the corpora haemorrhagica and corpora lutea at the middle stage of pseudopregnancy. These results were agreeing with findings in human (Sasano and Suzuki, 1997; Vazquez *et al*., 1999; Maybin and Duncan, 2004), in monkey (Hild-Petito *et al*., 1988), in cattle (Rueda *et al*., 2000; D'Haeseleer *et al*., 2007) and in rabbit (Parillo *et al*., 2013; Abd-Elkareem, 2017a). Our results were disagreeing with findings in pseudopregnant rabbits (Korte and Isola, 1988) as in this study the corpora lutea of pseudopregnant rabbits contained small amounts of PRA. Progesterone receptors in the large lutein cells and endothelial cells of the corpora haemorrhagica and corpora lutea are required for luteinization, maintenance of luteal structure and function (Duffy *et al*., 1997) and they regulate vascularization of the corpus luteum (Vazquez *et al*., 1999). The main function of the corpus luteum is secretion of the hormone P4 which is required for maintenance of normal pregnancy via PRA. The stroma cells surrounding both follicles and corpora lutea were stained positive for PR (Revelli *et al*., 1996).

The present study revealed that at the end of pseudopregnancy (18 days), the regressed corpora lutea of the pseudopregnant rabbit showed strong PRA immunostaining in the small lutein cells while the apoptotic large lutein cells showed negative reactivity. This agree with our results in 10 days post parturient rabbit ovary (Abd-Elkareem, 2017a).

The present study showed that the PRA positive small lutein cells of the regressed corpus luteum and the PRA positive proliferated theca interna cells of the atretic antral follicles were transformed into PRA positive interstitial gland cells. PRA may play a role in this transformation (Abd-Elkareem, 2017a). Interstitial gland cells of the rabbit ovary had two main sources. The first one was the theca interna cells of the atretic antral follicles and the second source was small lutein cells of regressed corpora lutea which originated from the theca interna cells of postovulatory follicle (Abd-Elkareem, 2010; Abd-Elkareem, 2014). This explain why the interstitial gland was well developed in the rabbit ovary especially after pregnancy or pseudopregnancy and its presence is essential for full reproductive capacity; maturation of the rabbit ovary and ovulation (Abd-Elkareem, 2014). Interaction between the interstitial gland cells and the lutein cells via progesterone receptor was necessary for progestational changes in the uterus of pseudopregnant rabbits (Abd-Elkareem, 2017b).

Functional corpora lutea were classified into Corps progestatifs (progestational corpora lutea) and: Corps gestatifs (gestational corpora lutea) (Dubreuil, 1962). Progestatifs are usually not as large nor do they secrete as much progesterone as do those of pregnancy (Greenwald and Rothchild, 1968). The main function of the corpus luteum (CL) is secretion of the hormone progesterone (P4) which is required for maintenance of normal pregnancy. The placenta of rabbit secretes low or physiologically insignificant quantities of progesterone, therefore the rabbits depend on their corpora lutea as the major source of progesterone throughout gestation and the corpora lutea must remain steroidogenically active throughout gestation (Keyes and Gadsby, 1987; Dharmarajan *et al*., 2004; Abd-Elkareem, 2017a). The corpus luteum is playing an important role in pregnancy maintenance of the rabbit and appears to require two luteotropins: estrogen from ovarian follicles and a placental luteotropic factor (Gadsby *et al*., 1983).

If the young embryo is to survive, the placenta of rabbit must transmit a signal that in some way prevent luteal regression, reflected in declining serum progesterone values by 15- 18 days after ovulation in pseudopregnant animals (Keyes and Gadsby, 1987; Abd-Elkareem, 2014). During pregnancy, the rabbit conceptus can be modifying the luteal responsiveness to PGF-2α and prolongs the life span of the CL probably by production of luteotrophic and/or antiluteolytic factors. The presence of the conceptus is apparently not necessary for maintenance of the CL until maternal recognition of pregnancy on Day 12 post coitum. It is then that the CL of pseudopregnant rabbits begins to regress; luteolysis in pseudopregnant rabbits occur by increase in luteal responsiveness to PGF-2α (Marcinkiewicz *et al*., 1992). In pseudopregnancy the CL is functional for only 16-18 days (Abd-Elkareem, 2014).

Progesterone acts as antiapoptotic factor in the CL by a PR-dependent mechanism. Decreased progesterone and progesterone receptor promote apoptosis in bovine luteal cells (Rueda *et al*., 2000). Prostaglandin F2 alpha (PGF2α) regulates luteolysis in rabbit by down-regulation of estrogen receptors and modulation of pro- and anti-apoptotic pathways in CL (Maranesi *et al*., 2010). Rabbits PGF2α is either of uterine (Maranesi *et al*., 2010) or of luteal origin (Zerani *et al*., 2007). PR may be disappeared or decreased in the lutein cells either by the direct action of the PGF2α in the lutein cells and /or indirect way by apoptosis (Sakumoto *et al*., 2010). No steroid hormone receptors were expressed in the corpus albicans (Sasano and Suzuki, 1997). In conclusion progesterone receptor alpha (PRA) is the key of the progestational changes in the ovary and uterus of pseudopregnant rabbits.
